# RETRACTION: The Essential Oils of *Rhaponticum carthamoides* Hairy Roots and Roots of Soil‐Grown Plants: Chemical Composition and Antimicrobial, Anti‐Inflammatory, and Antioxidant Activities

**DOI:** 10.1155/omcl/9893569

**Published:** 2026-07-30

**Authors:** Oxidative Medicine and Cellular Longevity

RETRACTION: E. Skała, P. Rijo, C. Garcia, et al., “The Essential Oils of *Rhaponticum carthamoides* Hairy Roots and Roots of Soil‐Grown Plants: Chemical Composition and Antimicrobial, Anti‐Inflammatory, and Antioxidant Activities,” *Oxidative Medicine and Cellular Longevity*, 2016, 8505384, https://doi.org/10.1155/2016/8505384.

The above article, published online on 18 December 2016 in Wiley Online Library (wileyonlinelibrary.com), has been retracted by agreement between the Journal Editor‐in‐Chief, Jeannette Vasquez Vivar; and John Wiley & Sons Ltd. The retraction has been agreed due to concerns that have been raised with Figure 3b, also reported on PubPeer [1]. Specifically, there are multiple instances of highly similar bands shown in Figure 3a, between both the same and different conditions.

The authors responded to the concerns; however, due to the time elapsed since the study, they could not provide the original images for this figure. After assessing the response to the concerns, we conclude that neither the results nor the conclusions can be considered reliable, and the article is therefore retracted.

The authors disagree with the retraction.
